# Mineral Carbonation for Carbon Sequestration: A Case for MCP and MICP

**DOI:** 10.3390/ijms26052230

**Published:** 2025-03-01

**Authors:** Samantha M. Wilcox, Catherine N. Mulligan, Carmen Mihaela Neculita

**Affiliations:** 1 Department of Building, Civil and Environmental Engineering, Concordia University, Montréal, QC H3G IM8, Canada; wilcox.samantha@gmail.com; 2 Research Institute on Mines and the Environment (RIME), University of Quebec in Abitibi-Témiscamingue, Rouyn-Noranda, QC J9X 5E4, Canada; carmenmihaela.neculita@uqat.ca

**Keywords:** mineral carbonation, carbon sequestration, carbon dioxide, microbially induced carbonate precipitation, urease, carbonic anhydrase, microbial carbonate precipitation

## Abstract

Mineral carbonation is a prominent method for carbon sequestration. Atmospheric carbon dioxide (CO_2_) is trapped as mineral carbonate precipitates, which are geochemically, geologically, and thermodynamically stable. Carbonate rocks can originate from biogenic or abiogenic origin, whereby the former refers to the breakdown of biofragments and the latter precipitation out of water. Carbonates can also be formed through biologically controlled mechanisms (BCMs), biologically mediated mechanisms (BMMs), and biologically induced mechanisms (BIMs). Microbial carbonate precipitation (MCP) is a BMM occurring through the interaction of organics (extracellular polymeric substances (EPS), cell wall, etc.) and soluble cations facilitating indirect precipitation of carbonate minerals. Microbially induced carbonate precipitation (MICP) is a BIM occurring via different metabolic pathways. Enzyme-driven pathways (carbonic anhydrase (CA) and/or urease), specifically, are promising for the high conversion to calcium carbonate (CaCO_3_) precipitation, trapping large quantities of gaseous CO_2_. These carbonate precipitates can trap CO_2_ via mineral trapping, solubility trapping, and formation trapping and aid in CO_2_ leakage reduction in geologic carbon sequestration. Additional experimental research is required to assess the feasibility of MICP for carbon sequestration at large scale for long-term stability of precipitates. Laboratory-scale evaluation can provide preliminary data on preferable metabolic pathways for different materials and their capacity for carbonate precipitation via atmospheric CO_2_ versus injected CO_2_.

## 1. Introduction

Global warming and climate change have been significant concerns to scientists, engineers, and policy makers for a long time. The first Intergovernmental Panel on Climate Change (IPCC) was held in 1988 and the notorious Paris Agreement in 2015, which involved a commitment by 195 countries to limit global warming to 1.5–2 °C. Today, despite efforts to reduce greenhouse gas (GHG) emissions, global warming is projected at 3.5 °C by 2100 [1]. There is a need for carbon sequestration strategies that transform and sequester GHGs (CO_2_, methane (CH_4_) and fluorinated gases (hydrofluorocarbons (HFCs), perfluorinated compounds (PFCs), sulfur hexafluoride (SF_6_), and nitrogen trifluoride (NF_3_)) from the atmosphere, which, left untreated, create a warming effect.

Biological carbon sequestration can utilize plants and microorganisms (bacteria, fungi, archaea, cyanobacteria, and algae) to fix inorganic CO_2_ as organic products (cellulous, lignocellulose, chitin, hemicellulose, lignin, etc.) or mineral precipitates for carbon capture and utilization [2]. Some microorganisms (carboxydotrophs) are carbon dependent and utilize atmospheric CO and CO_2_ as their energy source [3]. Further, there are many identified pathways and enzymes (e.g., CA, RuBisCO, carbon monoxide dehydrogenase (CODH), etc.) linked to biologic carbon sequestration [2]. Mineral carbonation, specifically, biogenic mineral carbonation, offers a promising opportunity to sequester atmospheric CO_2_ through naturally occurring processes. The process can be applied passively (trapping atmospheric CO_2_) or as a carbon capture and storage (CCS; actively injecting CO_2_) strategy.

While microbially induced carbonate precipitation (MICP) is a well-studied biological technique for soil and cement strengthening and restoration of construction materials, limited research has evaluated its feasibility as a carbon sequestration technique. In short, the microorganisms act as a catalyst to chemical precipitation and capture carbon as mineral carbonate precipitates. These reactions can be naturally occurring or engineered to enhance or optimize precipitation and therefore carbon sequestration. Alternatively, microbial carbonate precipitation (MCP) is a passive precipitation technique driven by the organic material in the environment. The objective of this paper is to evaluate abiotic and biotic carbonation, making a case for MICP and MCP as viable mineral carbonation techniques for carbon sequestration.

## 2. Biochemical Precipitation

### 2.1. Carbonate Precipitates

Chemical precipitation is a complex process used for separation of solid substances from solution [4,5,6]. For precipitation to occur, the solute concentration must exceed the liquid–solid equilibrium of the solution, meaning it is in a supersaturated state [5]. Supersaturation is, therefore, the driving force of precipitation [4,5]. Thermodynamically, the Gibbs free energy (G) and the solubility product constant (K_sp_) govern the reaction equilibria and the solubility equilibria, respectively [7].

Kinetically, there are three main processes that direct precipitation. These include nucleation, growth, and agglomeration [5]. Nucleation refers to the birth of particles via condensation of ions. It can be homogenous (spontaneous) or heterogenous (prompted by foreign particles) [5,7]. Growth and agglomeration refer to the enlargement of particles. Crystal growth refers to the enlargement from material deposition onto formed particles via transport to the crystal surface, adsorption onto the crystal surface, or formation of crystal lattice bonds [7]. Agglomeration refers to the contact of two or more particles, which over time forms a stable particle [5]. The supersaturation will impact the type of nucleation and growth, which affects the texture and purity of the crystals [5]. Precipitates can also undergo aging (re-arrangement of the crystal structure to form larger, pure crystals with time) and coprecipitation (ion inclusion into the crystal structure or adsorption onto the crystal surface) [7]. Chemical precipitation factors include the soil–water system, pH (favors high pH), Eh, type and concentration of metals and metalloids (metal(loid)s), dissolved organic carbon (DOC), and inorganic and organic ligand presence [8].

Carbonate rocks can exist as igneous [9], sedimentary [10], and metamorphic rocks [11]. However, they primarily present as sedimentary rocks, either as limestone (CaCO_3_; calcite and aragonite) or dolostone (CaMg(CO_3_)_2_) [12]. However, minerals are distinct from rocks, and there are numerous carbonate minerals: magnesite (MgCO_3_), siderite (FeCO_3_), dolomite (CaMg(CO_3_)_2_), otavite (CdCO_3_), rhodochrosite (MnCO_3_), cerussite (PbCO_3_), smithsonite (ZnCO_3_), strontianite (SrCO_3_), witherite (BaCO_3_), etc. Calcium carbonate is the most abundant of the carbonate minerals, occurring as calcite, aragonite, and vaterite anhydrous polymorphs [10,12], whereby calcite is the most thermodynamically stable and vaterite is the least. Less reported are the hydrous (monoclinic ikaite (CaCO_3_·6H_2_O) and calcium carbonate monohydrate (CaCO_3_·H_2_O)) and amorphous (ACC–CaCO_3_·nH_2_O) polymorphs [12].

The crystal structure and morphology of carbonate minerals varies significantly. Calcite and dolomite typically have a hexagonal crystallography, whereas aragonite is usually an orthorhombic structure [13,14]. However, the degree of supersaturation has been shown to alter the crystal form [15]. This is in conjunction with morphologic changes in the minerals due to crystal growth rate dependent on supersaturation [12,16]. Therefore, the solubility of carbonate minerals is essential to crystal formation. The reversible reaction for CaCO_3_ dissolution and precipitation is shown in Equation (1), where the rightward reaction illustrates the dissolution of carbonate minerals, and the leftward reaction demonstrates precipitation [12]. Relative solubility is impacted by crystal size, heterogeneity, defects, porosity, and organic matrices, with mineralogy the most significant [12]. Magnesium (Mg^2+^), for example, is known to inhibit calcite growth [16,17,18] and increase calcite solubility [12]. Furthermore, CO_2_ concentration impacts solubility and precipitation, in which an increase in CO_2_ also increases CaCO_3_ solubility, causing dissolution [12,19]. Temperature and pressure also influence calcite formation, since temperature and pressure increases can cause CaCO_3_ solubility decreases, which favors precipitation [12,19,20]. It should be noted that CO_2_ solubility is inversely correlated to temperature, and positively correlated to pressure [21].

(1) $${CaCO}_{3}+H_{2}O+{CO}_{2}↔{Ca}^{2+}+2{HCO}_{3}^{-}$$

Carbonate minerals form carbonate rocks through deposition and diagenesis [12]. They are formed in marine (ocean, sea (neritic and pelagic)) and terrestrial (lakes, hot and cold springs, caves, soils) environments, originating from biogenic, abiogenic, or complex mixtures of both components [12]. Carbonate sediments can be categorized as deep-sea oozes, carbonate turbidites, shelf accumulations of lime sands, silts, muds, organic reefs, and reef debris [22]. They are classified according to their compositions, fabric, and origin [23]. The biosphere and depositional environment impact the skeleton mineralogic, petrographic, and geochemical vestiges of the carbonate rocks [12]. Biogenic deposition often refers to the breakdown of invertebrate biofragments (i.e., shells, single cells, colonial skeletons) and crystallites within algal tissue or the calcification of microbes (i.e., calcimicrobes), whereas abiogenic deposition results from precipitation out of seawater or freshwater [12]. Diagenesis is a complex process encompassing 30 processes, including lithification destructive processes, re-crystallization, and grain-diminution [24]. In Precambrian times, carbonate rocks originated from algae pH control in lagoons and direct chemical precipitation out of sea water, while Cambrian origin often resulted from the organisms extracting carbonate out of sea water [25].

### 2.2. Biogenic Carbonate Precipitates

Bioprecipitation can encompass the formation of all biologically facilitated crystalline or amorphous precipitates with both organic and/or inorganic components. Bioprecipitation uses microorganisms to catalyze chemical precipitation reactions. It can incorporate different microorganisms to facilitate distinct metabolic pathways. The aim is to precipitate compounds (i.e., carbonates, hydroxides, phosphates, sulfides, sulfates, arsenates, silicas, chlorides, fluorides, oxides, oxalates, etc. [26,27,28,29]) with low solubility.

Abiotic precipitation varies significantly from biotic precipitation. The morphology is a key indicator used to distinguish inorganic and abiotic processes from biogenic minerals [30], which are typically differentiated by their unusual external morphology [29]. An interesting characteristic of biominerals is the composites or agglomeration of crystals separated by organic material [29]. Researchers have found differences in shape, size, crystallinity, isotopic, trace compositions, organic functional groups, activation energy, and enthalpy between biotic and abiotic precipitates [29,30]. Within lacustrine systems, for example, carbonate minerals are typically of biological origin or from direct biological activity, whereby the oxic (bio-induced pelagic CO_3_^2−^ precipitates), suboxic, and anoxic (microbial-induced diagenetic CO_3_^2−^ precipitates) conditions in the microenvironment with the ion supply impact the microbial pathway (see Section 2.4) [31]. Therefore, microenvironmental conditions impact the carbon isotopic composition of dissolved inorganic carbon (DIC) in pore water and the carbon isotopic composition of precipitates [31]. Further differences have been reported between biotic, organogenic (nutrient composition without bacterial cells), and inorganogenic (chemical reaction) forms of CaCO_3_ precipitation, suggesting thermal stability is highest in biotic calcite [30]. The calcite crystal growth rate and biotic growth rate in carbonate deposits will influence whether it is biotic/abiotic [32]. If the supersaturation state is high, calcite will favor abiotic precipitation, as crystal formation outpaces microbial growth rates [32]. In the context of carbon sequestration, abiotic factors regulating sequestration include pH and medium components (i.e., urea), while biotic factors are dependent on the species or strains [33]. Again, resident biota will impact the CO_2_ levels inducing dissolution (water absorption of CO_2_ respiration in soil increasing acidity and dissolution) or precipitation (removal of CO_2_ in seawater via phototrophs during photosynthesis) [12]. The degree of control exerted by the microorganism will dictate the biological mechanism occurring [29].

**Figure 1:**
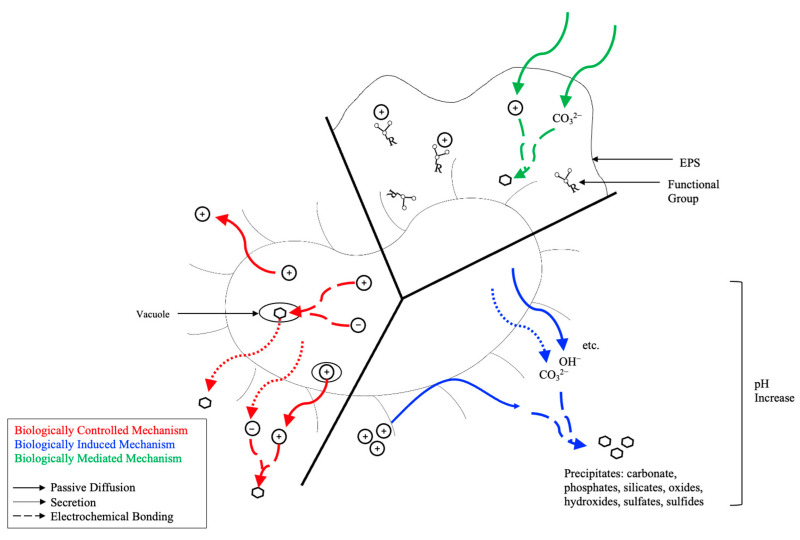
Biochemical precipitation mechanisms, including biologically controlled mechanisms (**left**), biologically induced mechanisms (**right**), and biologically mediated mechanisms (**top**). The red lines represent passive diffusion, blue lines represent active pumping, and green lines represent secretion. Adapted from [33,36,45].

The interaction between microbial activity, the external environmental conditions, and the overall biofilm matrix will determine how and whether biotic precipitates form. There are three primary mechanisms (Figure 1 and Table 1) capable of facilitating bioprecipitation, including biologically controlled mechanisms (BCMs), biologically induced mechanisms (BIMs), and biologically mediated mechanisms (BMMs; otherwise termed biologically influenced mechanisms). Under certain conditions, microorganisms can synthesize minerals via nucleation and growth facilitating BCM [26,27,29,34,35]. Cellular activities including active pumping, passive diffusion, and secretion can lead to precipitation of particles in the extracellular, intracellular, or intercellular environment [26,27,29]. The final resting place of precipitates is within or on the microbial cell [27,34,36]. The composition, morphology, and localization of precipitates are influenced by the species-specific process [26,29]. BIMs involve the metabolic activity of the microorganism, which interact with the environment to facilitate precipitation [26,27,29,36]. The precipitates form in the extracellular environment, where nucleation and growth typically transpire on the microbial cell wall [29]. Precipitation is dependent on the environmental conditions (i.e., pH, redox potential, CO_2_, etc.) and the subsequent supersaturation [29,34,35,37]. The composition, particle size, crystal purity, and morphology are varied due to diverse environmental conditions [26,29]. Passive precipitation is caused by the BMM due to the interaction of organic matter (i.e., extracellular polymeric substances (EPS), biofilm, and the organic/inorganic compounds) within the matrix [26,34]. Biological activity does not directly cause precipitation.

Both BIM and BMM utilize prokaryotes to facilitate precipitation. While the presence of microorganisms is not directly required for BMM, the organic EPS matrix is an extension of the microbial cell [38], as shown in Figure 1. Mineral deposits via BIM and BMM can be classified as stromatolites, thrombolites, and leiolite [38]. For carbonate precipitation, the BIM and BMM are referred to as MICP and MCP, respectively. Table 2 provides a comparative analysis of these methods. For global carbon sequestration, MCP is well established as a long-term storage technique for carbon. Carbon can be trapped in terrestrial environments (i.e., soils, caves, deserts, tundra, boreal forests, temperate forests, tropical forests, grasslands, etc.) in the organic matrix of soil as soil organic carbon (SOC), carbonate deposits (precipitated via plants, fungi, or bacteria), and/or vegetation [38,39,40]. Plants are also able to store inorganic CO_2_ through biological carbon mitigation as organic carbon through photosynthesis [41]. As a byproduct of photosynthesis, plants can precipitate whewellite (Ca(C_2_O_4_)·H_2_O) storing atmospheric CO_2_ [38]. Liu et al. [42] found that EPS and EPS-carbon are positively correlated to SOC, whereby EPS-carbon accounts for ≤10.69% the total organic carbon in surface sediments. This is quite significant, since 75% of organic carbon is sequestered in mangrove ecosystems in sediments [42,43]. MICP, however, is less established in terms of global carbon sequestration. MICP is shown to store carbon as carbonate deposits in marine environments, hypersaline lakes, freshwater environments, and continental environments [38], which act as carbon sinks for carbon sequestration.

**Table 1:** Comparative analysis of BCM, BIM, and BMM. Adapted from [38,44].

Mechanism	Precipitate Location	Conditions	Organisms	Level of Organism Control	Precipitated Minerals
BCM	Intracellular, intercellular, extracellular	Controlled by cellular activities	Eukaryotes	High	Magnetite, greigite, amorphous silica, calcite
BIM	Extracellular	Reactive surfaces & metabolism	Prokaryotes	Moderate	Iron hydroxides, magnetite, manganese oxides, clays, amorphous silica, carbonates, phosphates, sulfates, sulfide minerals
BMM	EPS matrix	Alkalinity engine & organic matter	Not required	Low	Carbonate minerals

**Table 2:** Comparative analysis of MICP and MCP.

Carbonate Precipitation	Mechanism	Microbial Involvement	Application	Research Topics	Advantages	Drawbacks
MICP	BIM	Active	In situ ^1^ & ex situ ^2^	Restoration of calcareous stones & construction materials, soil strengthening, selective plugging for oil recovery, bio-clogging, soil thermal conductivity, dust suppression, erosion control, liquefaction mitigation, wastewater treatment, bioremediation, CO_2_ sequestration [45]	Wide range of applicable microorganisms, applicable to a wide range of environments, low costs, high CaCO_3_ conversion, short timeframes [45]	Potential for harmful byproducts, bio-clogging at injection site, requires specific conditions
MCP	BMM	Passive	In situ ^1^ & ex situ ^2^	Wastewater treatment, oil recovery, biofilm barriers, bioremediation [46]	Wide range of environments, adaptable to versatile environmental conditions	Variable efficacy for carbonate precipitation, slower rates of precipitation

^1^ In situ biostimulation, ex situ biostimulation and bioaugmentation [47]. ^2^ Material pre-treatment [35].

It should be noted that in addition to bacterial carbonate precipitation, eukaryotes (i.e., *coccolithophores* and *foraminifera*) can precipitate carbonates to form shells or skeletons via BCM [44,48]. The formation of CaCO_3_ exoskeletons plays an important role in the carbon cycle, impacting CO_2_ flux in seawater and inorganic carbon transport in oceans and sediments [48]. *Coccolithophores*, specifically, are promising for global climate change because (i) they are phytoplankton (autotrophic plankton obtaining energy through photosynthesis); (ii) they produce dimethyl sulfide (DMS), creating albedo effects via formation of highly reflective clouds; (iii) they control CO_2_ influx into water via precipitation of CaCO_3_, which depletes dissolved bicarbonate (HCO_3_^−^), increasing dissolved CO_2_ [44,48,49]. However, increased CO_2_ has been shown to decrease CaCO_3_ precipitation in marine phytoplankton [50], since increased atmospheric CO_2_ also increases carbonic acid (H_2_CO_3_), producing more HCO_3_^−^ and H^+^ ions (Equation (2)), which dissolves CaCO_3_, decreasing pH [48]. Furthermore, there are numerous autotropic carbon-fixation mechanisms identified in archaea, including the Calvin cycle, reductive citric acid cycle, reductive acetyl-coenzyme A pathway, 3-hydroxypropionate bicycle, hydroxypropionate-hydroxybutyrate cycle, and dicarboxylate-hydroxybutyrate cycle [51].

(2) $${CO}_{2}+H_{2}O↔H_{2}{CO}_{3}↔{HCO}_{3}^{-}+H^{+}↔{CO}_{3}^{2-}+{2H}^{+}$$

### 2.3. Microbial Carbonate Precipitation (MCP)

The precipitation of CaCO_3_ is dependent on the calcium (Ca^2+^) concentration, DIC, nucleation sites, and pH [27,52]. For the purpose of microbial precipitation, the principal role of bacteria is to create an alkaline environment via pH and DIC increase [52]. This can occur as a BMM (passive process) or BIM (active process) [26].

Biologically mediated CaCO_3_ precipitation occurs from the interaction of EPS and Ca^2+^ ions. As mentioned previously, the process does not require direct biological activity but is influenced by the organics associated with the cell wall and/or the EPS [26,34,37,53]. The microorganisms secrete natural polymers (polysaccharides, lipids, proteins, etc.), forming an organic matrix. These organic polymers favor heterogenous nucleation, leading to stabilization of new particles [37]. They can also act as nucleation sites [53,54]. Further, an increase in pH causes functional groups to deprotonate, causing exopolysaccharides produced by the microbial cell to have an overall negative charge and bind to metal(loid) ions [26].

The bacterial cell surface and biomass surfaces have an electronegative charge due to the presence of carboxyl, phosphoryl, amino, and sulfo groups [55]. The negative surface charge allows redox processes, adsorption, complexation, ion exchange, electrostatic attraction, and precipitation to immobilize metal(loid)s in situ [27,56]. For example, adsorption of positive divalent cations to the bacterial cell wall can lead to the precipitation of carbonates [27]. Initial adsorption of bacteria onto mineral surfaces is governed by hydrophobicity and electrostatic forces, while final attachment of bacteria to minerals is influenced by biofilm formation and secretion of exudates [57]. Therefore, uptake of cationic metal(loid)s can create a state of oversaturation in the microenvironment, leading to precipitation [58]. Further, both the cell wall and EPS have metal binding capacities; however, fate transport of these bonded metals is not known [59]. The bacterial cell wall provides nucleation sites for mineral deposition of biological precipitates [60,61]. This is in addition to exopolymers, biofilms, and inactive spores, which also provide sites for nucleation [26].

The function of EPS is important to the carbonate precipitation process. EPS are high-molecular-weight natural polymers (i.e., lipids, proteins, polysaccharides, DNA, etc.) secreted by autotrophic and heterotrophic microorganisms, responsible for the functionality and structural integrity of the biofilms [58,62]. The macromolecules (through dispersion forces, electrostatic interactions, and hydrogen bonds) create a gel-like substance around the cells, establishing a stable consortia of microorganisms [62]. Similar to the bacterial cell surface, EPS contains functional groups, including carboxyl, phosphoryl, amino, and hydroxyl groups [58,63,64]. These negative functional groups can attract positive divalent cations, thereby promoting precipitation of metal carbonate (MCO_3_) compounds through local alkalization or inhibiting precipitation by removing the free cations from solution and reducing saturation. If the latter transpires, MCO_3_ can be precipitated out of solution when EPS degrades and saturates solution with metal divalent cations [58,63]. The biochemical composition of EPS can affect the resulting mineralogy of CaCO_3_, altering the polymorph (aragonite, vaterite, calcite) [65]. It can also alter the crystal morphology of CaCO_3_ precipitates [66,67]. EPS is thought to influence the biofilm, cell adhesion, and capturing CaCO_3_ precipitates [54]. Both EPS and biofilm formation can reduce pore space, increase ductility, increase strength, reduce hydraulic conductivity, and reduce permeability [68].

### 2.4. Microbial-Induced Carbonate Precipitation (MICP)

**Figure 2:**
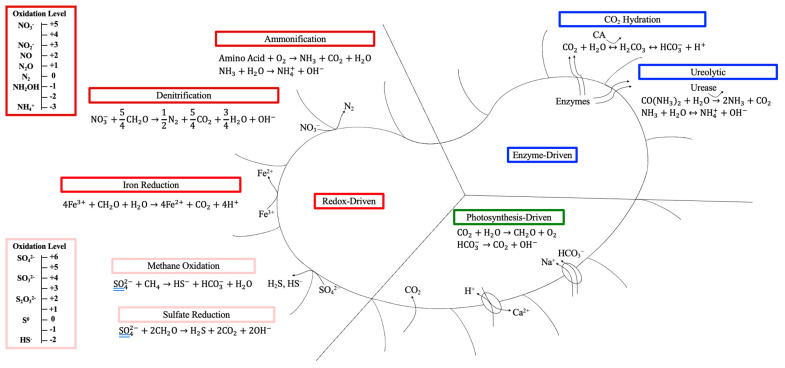
Biochemical reactions facilitating the different metabolic pathways of MICP, including redox-driven reactions (red), enzyme-driven reactions (blue), and photosynthesis-driven reactions (green). The reactions involved in the nitrogen (dark red) and sulfur (light red) cycle are highlighted. Adapted from [58,70].

MICP is a complex process involving numerous metabolic pathways. These metabolic pathways can be enzyme-driven, redox-driven, or photosynthesis-driven reactions (Figure 2) [28]. Equations (3) and (4) illustrate the governing equations for calcium carbonate precipitation induced by biological processes [69]. The Ca^2+^ ion can be interchanged by other divalent cations to precipitate other MCO_3_ compounds [45].

(3) $${Ca}^{2+}+Cell\to Cell-{Ca}^{2+}$$

(4) $$Cell-{Ca}^{2+}+{CO}_{3}^{2-}\to Cell-{CaCO}_{3}$$

Since SOC is considered energy limiting for microbial growth and carbonate mineralization [71], a nutrient broth (NB) is often added to supplement nutrient-deficient substrates for microbial growth. It is a complex concoction of chemicals and nutrients dependent on the desired microbial pathway. Nutrients often include carbon, nitrogen, and phosphorous, whereby an optimized ratio (C:N:P) is analyzed, typically approximately 100:10:1 [72]. The enzyme activity of urease, phosphatase, and dehydrogenase is involved in biogeochemical cycles of phosphate, nitrogen, and oxidation reduction of organic compounds and can illustrate the fertility of substrates [73].

Essential to MICP is a Ca^2+^ source. As mentioned above, Ca^2+^ concentration plays a vital role in CaCO_3_ precipitation. Typically, a calcium source is added to facilitate precipitation. The most common calcium source used in research is calcium chloride (CaCl_2_). The CaCl_2_ compound undergoes dissolution, and the Ca^2+^ ions precipitate CaCO_3_, while the chloride ions (Cl^−^) form ammonium chloride (NH_4_Cl), shown in Equation (5) [27,74]. However, calcium acetate (C_4_H_6_CaO_4_) and calcium nitrate (Ca(NO_3_)_2_) are also used to facilitate carbonate precipitation [27]. Enhanced MICP is linked to higher concentrations of urea and CaCl_2_ [75]. Stoichiometric calculations are required to determine the amount of urea necessary to convert all Ca^2+^ to CaCO_3_ [76].

(5) $${Cl}^{-}+{HCO}_{3}^{-}+{NH}_{3}\to {NH}_{4}Cl+{CO}_{3}^{2-}$$

**Figure 3:**
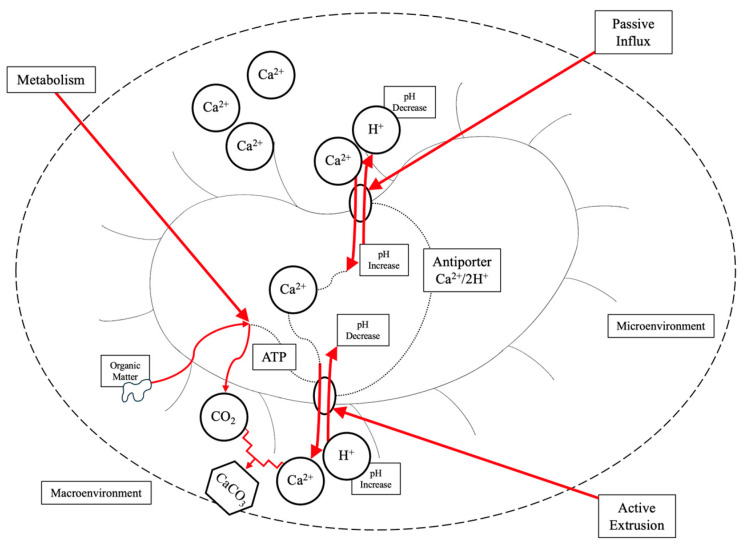
Calcium regulation in microorganisms showcasing the interaction and metabolism of calcium, leading to CaCO_3_ precipitation. Adapted from [52].

The interaction of calcium with the microbial cell is an intricate process. The microorganism is surrounded by a thin layer of water, and when subjected to a low Reynold’s number, protons (pH), DIC, and Ca^2+^ can concentrate in the microenvironment [52]. Ca^2+^ accumulates outside the microbial cell wall and is not likely utilized by the microbial metabolic processes [60]. McConnaughey and Whelan [77], Castanier [78], and Hammes and Verstraete [52], among other researchers, characterize the difference between “active precipitation” and “passive precipitation”, whereby the former is linked to ion transport and exchange (specifically Ca^2+^) through the cell membrane, and the latter encompasses precipitation via the metabolic pathways discussed below. Active precipitation is governed by calcium regulation via influxes and outflows (Figure 3). The transport mechanisms enabling active precipitation can be further classified as active or passive. Ca^2+^ influxes can be a passive transport mechanism based on the electrochemical gradient [52]. Concentrations of Ca^2+^ in the extracellular environment are typically 1000 times greater than in the intracellular environment due to low permeability of the cell envelope, high buffering capacities, and effective export mechanisms [79]. The passive transport mechanisms include antiporters (Ca^2+^/2H^+^, Ca^2+^/2Na^+^, etc.), protein-based channels, and non-proteinaceous channels. Active transport, however, includes Ca^2+^ transport against the electrochemical gradient using ATP-energy and ATP-dependent pumps [52].

The metabolism of calcium by the microorganism is a driving factor for CaCO_3_ precipitation. In the microenvironment, when Ca^2+^ concentrations and pH (low H^+^ proton concentration) are high in comparison to the microbial intracellular environment, the difference in electrochemical gradient will cause the Ca^2+^/2H^+^ antiporter to accumulate Ca^2+^ in the microbial cell and release H^+^ protons to the extracellular environment. Through active extrusion, the microorganism will then release Ca^2+^ through ATP-dependent calcium pumps and uptake H^+^ protons. This will create localized alkaline conditions and high Ca^2+^ concentrations ideal for precipitation. The metabolism of organic matter is required for ATP, which releases DIC to the extracellular environment in the form of CO_2_. The CO_2_ will undergo hydrolysis to form HCO_3_^−^ and CO_3_^2−^ ions (Equation (6)), which will interact with the Ca^2+^ ions and precipitate CaCO_3_. This will impact the CaCO_3_ solubility product. As the soluble Ca^2+^ ions decrease and there is an increase in acidity, the conditions become favorable for bacterial proliferation [52].

(6) $${Ca}^{2+}+{HCO}_{3}^{-}\to {CaCO}_{3}+H^{+}$$

#### 2.4.1. Nitrogen Cycle

The nitrogen cycle plays a significant role in MICP. Three of the MICP metabolic pathways rely on the nitrogen cycle: denitrification, ammonification, and urea hydrolysis. In each of these scenarios, a pH increase (NH_4_^+^ and OH^−^) in the presence of Ca^2+^ ions lead to CaCO_3_ precipitation. Shown in Equation (2), a decrease in H^+^ ions shifts the CO_3_^2−^–HCO_3_^−^ equilibrium to its CO_3_^2−^ form, inducing CaCO_3_ precipitation [78].

Denitrification (Equation (7)) utilizes nitrate-reducing bacteria (NRB) to facilitate precipitation [58,80]. The dissimilatory reduction of nitrate (NO_3_^−^) increases pH through the consumption of NO_3_^−^ and the generation of OH^−^ ions [81]. Under anaerobic or hypoxic conditions, NO_3_^−^ acts as the electron acceptor producing inorganic carbon in the form of CO_2_ [58,81]. Nitrogen (N_2_) gas is an end product of dissimilatory nitrate reduction; however, intermediates include nitrite (NO_2_^−^), nitric oxide (NO), and nitrous oxide (N_2_O) [82]. Toxic intermediates (NO_2_^−^ and N_2_O) can accumulate if the involved enzymes are inhibited [58,83]. There are four enzymes involved in the denitrification process: nitrate reductase (Nar), nitrite reductase (Nir), nitric oxide reductase (Nor), and nitrous oxide reductase (Nos) [82]. The localization, lifetime, regulatory mechanisms, kinetics, and sensitivity to inhibitors are different for each of these enzymes, which can lead to incomplete denitrification at any of the reduction steps [83]. For example, nitrate or calcium overloading can cause inhibition, yielding the accumulation of toxic intermediates [83]. Synthesis of these enzymes is dependent on oxygen (O_2_) concentration, pH, and temperature [84]. Evidence suggests nitrate plays a role in carbon-fixing pathways during carbon sequestration via revegetation, whereby nitrate directly impacts soil labile organic carbon and indirectly influences carbon-fixing microorganisms [85]. Furthermore, microbial genes identified in soils are involved in the nitrogen cycle and carbon fixation [86].

(7) $${NO}_{3}^{-}+\frac{5}{4}{CH}_{2}O\to \frac{1}{2}N_{2}+\frac{5}{4}{CO}_{2}+\frac{3}{4}H_{2}O+{OH}^{-}$$

Ammonification (Equation (8)) utilizes amino acids to produce NH_3_ and CO_3_^2−^ via myxobacteria to induce precipitation [58]. *Myxococcus xanthus*, for example, is shown to induce precipitation of calcite and vaterite crystals [87,88,89]. This occurs under aerobic conditions with gaseous or dissolved oxygen and organic matter. These heterotrophic microorganisms use amino acids as an energy source and contribute to the degradation of organic matter [58,78]. The hydrolysis of NH_3_ produces hydroxide ions (OH^−^), creating a pH increase and leading to local supersaturation around the microbial cell, favoring precipitation [58]. Also formed from NH_3_ hydrolysis is the NH_4_^+^ byproduct, often present in an aqueous state. NH_4_^+^ can easily convert into NO_3_^−^ and NO_2_^−^ [90], which can cause the accumulation of toxic nitrogen species in the environment. In addition, NH_4_^+^ in surface water can promote toxic algal bloom growth, impacting fish, flora, and fauna [91].

(8) $$Amino\;Acid+O_{2}\to {NH}_{3}+{CO}_{2}+H_{2}O$$

Urea hydrolysis involves the degradation of urea via ureolytic bacteria. The process consists of three main stages: (i) urea hydrolysis; (ii) pH increase; and (iii) cementation [69]. Ureolytic bacteria required for urea hydrolysis have a direct impact on the concentration of DIC (cell respiration and the decomposition of urea) and pH within the environment [58]. As part of the urea hydrolysis reaction (Equations (9) and (10)), 1 mol of urea (CO(NH_2_)_2_) via hydrolysis produces 1 mol carbamic acid (NH_2_COOH) and 1 mol NH_3_, where 1 mol NH_2_COOH undergoes spontaneous hydrolysis to produce 1 mol NH_3_ and 1 mol carbonic acid (H_2_CO_3_) [26,27,92]. This occurs via secretion of the enzyme urease (urea amidohydrolase; E.C. 3.5.1.5; nickel-containing metalloenzyme), which acts as a biological catalyst [93,94]. The enzyme speeds up the chemical reaction by lowering the activation energy via low-energy enzyme–substrate (i.e., urea) complexes. The bacteria use urease to hydrolyze CO(NH_2_)_2_ (added to the NB; NBU) by increasing ambient pH and using CO(NH_2_)_2_ as a nitrogen and energy source [60,93]. The NH_3_ (Equation (11)) produced from enzymatic urea hydrolysis will again undergo hydrolysis to form NH_4_^+^ and OH^−^, which increases pH [26,27,92]. The pH increases via urease can create a localized alkaline state in the microenvironment around the microbial cell, leading to CaCO_3_ precipitation on or around the cell wall [60,74]. Urease production and subsequent CaCO_3_ precipitation are impacted by the temperature, pH, concentration of CO(NH_2_)_2_, concentration of NH_3_, carbon source, and incubation period [95].

(9) $${CO{(NH}_{2})}_{2}+H_{2}O\to {NH}_{2}COOH+{NH}_{3}$$

(10) $${NH}_{2}COOH+H_{2}O\to {NH}_{3}+H_{2}{CO}_{3}$$

(11) $${2NH}_{3}+{2H}_{2}O\to 2{NH}_{4}^{+}+2{OH}^{-}$$

Through the precipitation of CaCO_3_ is the development of cementation, which pertains to the large-scale precipitation in-between solid particles forming a biocement matrix [35,45]. It involves the dissociation of a calcium source into soluble Ca^2+^ ions [81], and an increase in CO_3_^−^ ions to reach the supersaturation state inducing CaCO_3_ precipitation [69]. Again, NH_4_^+^ is produced as a byproduct. It should be noted that urea is very stable, and the purely chemical breakdown is independent of the pH between 2 and 12 [96]. The non-enzymatic process decomposes urea via elimination of NH_3_ (half-life of 33 years at 25 °C) or spontaneous hydrolysis (half-life of 520 years at 25 °C) [97]. However, application of urease drastically increases the rate of reaction to a half-life in the microsecond range [97,98].

Several methods have been explored to reduce or remove harmful NH_4_^+^ byproduct from nitrogen-driven MICP pathways. These include flushing and extracting NH_4_^+^ with geophysical setups, electrokinetic retention of NH_4_^+^ in the cathode chamber of an electrokinetic cell, NH_4_^+^ precipitation via additives, and utilization of alternative metabolic pathways [99]. An alternative metabolic pathway is iron reduction utilizing iron-reducing bacteria [80,100]. Ferric iron (Fe^3+^) acts as an electron acceptor in the presence of a carbon source, reducing to ferrous iron (Fe^2+^) and CO_2_ (Equation (12)) [80]. However, mineral precipitates are unstable and easily impacted by other ions [47]. Current research on this metabolic pathway is limited, although application of ureolytic MICP to iron-based substrates is emerging [101,102]. Iron-reducing bacteria have shown an impact in the complex coupling of Fe and C affecting carbon sequestration in paddy soils [103]. Furthermore, Fe can trap SOC via adsorption, coprecipitation [104], whereby ~21.5% of SOC is bound to reactive Fe phases in sediments [105].

(12) $${CH}_{2}O+4{Fe}^{3+}+H_{2}O\to 4{Fe}^{2+}+{CO}_{2}+4H^{+}$$

**Figure 4:**
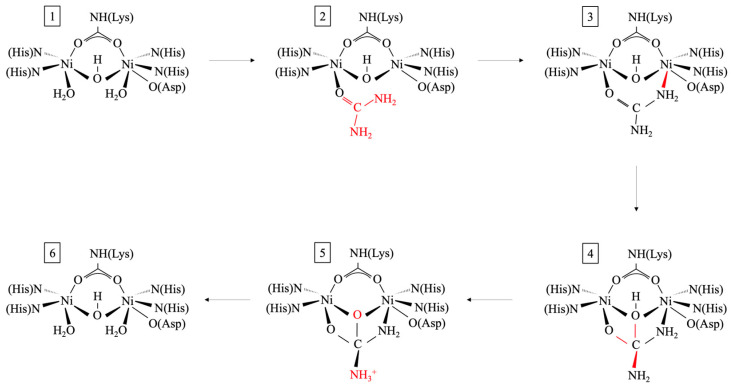
Simplified schematic of the structure-based reaction mechanism of urease. Red indicates the changes step by step. The flap is open (1), and urea enters the activated site, replacing water and binding to carboxyl oxygen (2). The flap closure enables urea binding to Ni^2+^ (3). The carbon atom on urea undergoes nucleophilic attack via Ni^2+^ bridging OH, creating a tetrahedral intermediate (4). The Ni bridging OH transfers the hydrogen atom to the distal urea NH_2_ group, forming NH_3_^+^ (5). The distal C-N bond is broken, and all products are released via a flap opening, which rehydrates the active site (6). Adapted from [108,111].

The efficacy of the urease enzyme can be impacted by nickel. The urease enzyme is composed of structural genes and accessory genes in operons and clusters [26,106]. Inactive urease (apo-urease) has structural genes (*ureA*, *ureB*, and *ureC*) requiring accessory genes (*ureD*, *ureF*, *ureG*, and *ureE*) for activation. Activation involves CO_2_ uptake for lysine carbamylation, hydrolysis of guanosine triphosphate (GTP), and Ni^2+^ delivery to its active site [107]. As mentioned preciously, nickel is incorporated in the active center of urease [26] and contains two nickel ions (Ni^2+^) bridged by a hydroxyl group and a carbamylated lysine [108]. The *ureE* gene, specifically, is responsible for delivering Ni^2+^ to the active site, leading to fully active urease (holo-urease) and subsequent urea hydrolysis [107]. A mobile flap (from the helix-turn-helix motif) covers the active site, restricting access [109]. As shown in Figure 4, ureolysis occurs when the flap is open and urea enters the active site, replacing water molecules bound to Ni^2+^ ions. The C-O bonds in urea are polarized and undergo nucleophilic attack because of the highly electrophilic Ni ions with the bridging OH [107,109,110]. The NH_2_ is protonated by the bridging Ni OH (or His320, Ala167, Ala363, Cys319, His219, G277) [110], and the C-N bond is broken, releasing NH_3_ [107,109]. The carbamate (CH_2_NO_2_^−^) remaining decomposes further (Equation (13)), and all products are released with the flap opening [107]. Furthermore, urease inhibition is attributed to Ni^2+^ binding, which leads to a loss in urease and catalytic activity. This includes impacts to the direct binding of Ni^2+^ at the active urease sites; covalent modifications that cover the Ni^2+^ center; and metal ion chelators that sequester Ni^2+^, thereby inhibiting the formation of the Ni^2+^ center [108]. Ni^2+^ in the form of nickel chloride (NiCl_2_) and nickel nitrate (Ni(NO_3_)_2_) has been added to the NB to enhance CaCO_3_ precipitation [111,112]. In addition to ureolysis, nickel is involved in hydrogen metabolism and methane biogenesis, and acts as an essential nutrient to microorganisms [109].

(13) $${{NH}_{2}COO}^{-}+2H_{2}O\to {NH}_{4}^{+}+{HCO}_{3}^{-}+{OH}^{-}$$

Application of ureolytic MICP for carbon sequestration showed efficacy ≤86.4%, dependent on the bacterial community structure and pH [113]. Higher-headspace CO_2_ uptake was shown with *Sporosarcina*, *Sphingobacterium*, *Stenotrophomonas*, *Acinetobacter*, and *Elizabethkingia* species [113]. An increase ≤ 148.9% in CO_2_ uptake through calcification can be shown in optimal urea growth media [33]. Conversely, ureolytic bacterial growth utilizing *Bacillus megaterium* demonstrated comparable quantities of precipitated CaCO_3_ with 99.5% pure CO_2_ influx to that of 2% NBU [114].

#### 2.4.2. Sulfur Cycle

The sulfur cycle also plays an interesting role in MICP (Equation (14)). In sulfate (SO_4_^2−^)-rich environments, sulfate-reducing bacteria (SRB) facilitate either dissimilatory or assimilatory sulfate reduction, producing hydrogen sulfide (H_2_S) or organic sulfur (S), respectively [115,116]. This reaction transforms organic carbon in the form of an energy source to HCO_3_^−^, and will release OH^−^ and increase alkalinity, leading to a supersaturation state and therefore the likelihood of CO_3_^2−^ precipitation [58]. SRB (comprising 87% *Halanaerobiaceae, Halobacteroidaceae, Enterobacteriaceae*) have been enriched from sediment samples for carbon sequestration, whereby ~53% of precipitated carbonate minerals are derived from CO_2_ headspace [117]. In this study, headspace pressure played an integral role in carbonate precipitation at ≤ 14.7 psi [117]. The SRB can also degrade EPS, releasing trapped Ca^2+^ into the environment, leading to CaCO_3_ precipitation [58]. Further, the H_2_S produced during the reaction may subsequently degas, increasing pH, increasing precipitation, or being utilized by bacteria [78]. However, the H_2_S can be highly toxic [81], and if not degassed or if unused by bacteria, it can cause pH to decrease and inhibit precipitation [78]. The process is most prominent under anaerobic or anoxic conditions rich in organic matter [26,78]. Sulfate reduction contributes ~36–50% carbon mineralization in anaerobic wetlands [118] and plays a significant role in stromatolite formation [119].

(14) $${SO}_{4}^{2-}+2{CH}_{2}O\to H_{2}S+2{CO}_{2}+2{OH}^{-}$$

Methanogens also utilize SO_4_^2−^ to induce methane oxidation [58]. Under anaerobic conditions, methane oxidation favors carbonate precipitation (Equation (15)), while aerobic conditions increase alkalinity, favoring carbonate dissolution (Equation (16)) [58,120]. Both scenarios can facilitate carbonate precipitation in the presence of a divalent cation source (i.e., Ca, Fe, Mg, Mn, Ba) [121]. However, interesting is the removal of CH_4_ emissions and its application as a methane sink offsetting GHG emissions [122]. The process transforms CH_4_ to a less toxic form and locks carbon as mineral precipitates. Furthermore, in situ anaerobic carbonate precipitation via methane oxidation can be subdivided into sulfate-dependent precipitation in shallow sediments or marine silicate weathering in deep sediments [121]. Unlike the other metabolic pathways that are solely heterotrophic, methane oxidation can be both an autotrophic [26,123] or heterotrophic process [124]. It can also use alternative electron sources to SO_4_^2−^, including NO_3_^−^, NO_2_^−^, Fe, Mn, and humic acid [120]. While methane oxidation in terms of MICP is often neglected in experimental research, a recent study has identified methanogenesis as a metabolic pathway in activated anaerobic sludge [99].

(15) $${SO}_{4}^{2-}+{CH}_{4}\to {HS}^{-}+{HCO}_{3}^{-}+H_{2}O$$

(16) $$2O_{2}+{CH}_{4}\to {CO}_{2}+2H_{2}O$$

#### 2.4.3. Photosynthesis

Purely autotrophic pathways (i.e., non-methylotrophic methanogenesis, anoxygenic photosynthesis, and oxygenic photosynthesis) utilize gaseous or dissolved CO_2_ from the atmosphere, respiration, or fermentation [78]. This CO_2_ acts as their carbon source to produce organic matter and, under Ca^2+^ rich environments, can favor CaCO_3_ precipitation [78]. Photolithoautotrophs, specifically, cyanobacteria, have been studied for their capacity for carbonate biomineralization [120], with their higher affinity for environment CO_2_ due to photosynthesis and CO_2_ fixation by the Calvin cycle [125]. Approximately 70% of carbonate rock in the history of Earth is contributed to cyanobacteria [126]. Cyanobacteria carbonate mineralization occurs in four steps: (i) CO_2_/HCO_3_^−^ uptake for photosynthesis; (ii) OH^−^ release; (iii) OH^−^ and HCO_3_^−^ reaction forming CO_3_^2−^; (iv) carbonate precipitation [127]. The CO_2_ (Equation (17)) enters the cell wall via diffusion or a symporter, where CO_2_ produces organic matter utilized by the microorganisms [58]. HCO_3_^−^ (Equation (18)) is transported from the extracellular environment into the cell membrane, which dissociates into CO_2_ and OH^−^ [26]. HCO_3_^−^ is the predominant form of inorganic carbon transported into the cell [127]. The OH^−^ ion is released from the cell to the extracellular environment, increasing pH, which again favors CaCO_3_ precipitation in Ca^2+^-rich environments [26,58]. The equilibrium reached between HCO_3_^−^ into the cell and the efflux of OH^−^ from the cell causes the alkalinization in the microenvironment around the cell [127]. CaCO_3_ nucleation occurs on the sticky cell walls of cyanobacteria, which aid in binding [128]. Furthermore, calcium metabolism can store Ca^2+^ within the cell membrane, precipitating CaCO_3_ intracellularly, or can release Ca^2+^ through the Ca^2+^/2H^+^ antiporter, precipitating CaCO_3_ extracellularly [58]. This process has been investigated more frequently due to the nature of its less harmful byproduct (CH_2_O). The absence of NH_4_^+^ or H_2_S make this pathway desirable. Naturally occurring photosynthesis-driven carbonate mineralization is shown in karstic environments to offset mine-related GHG emissions by ~20% [129,130].

(17) $${CO}_{2}+2H_{2}O\to {CH}_{2}O+O_{2}$$

(18) $${HCO}_{3}^{-}\to {CO}_{2}+{OH}^{-}$$

**Figure 5:**
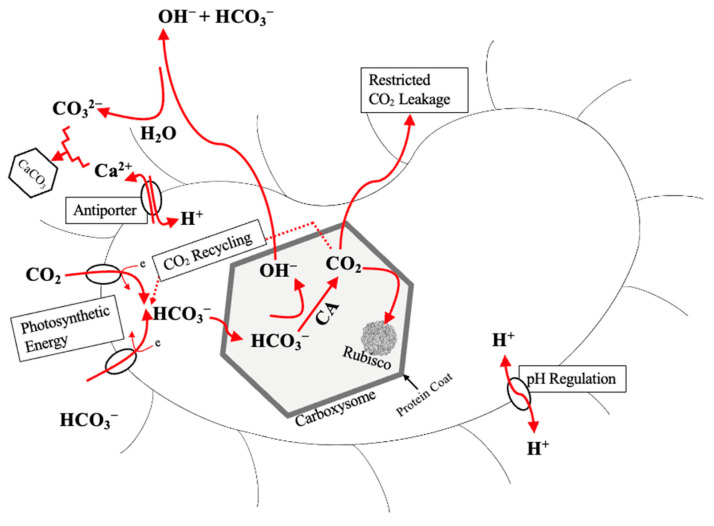
A generic model of the CCM of cyanobacteria showing accumulation of HCO_3_^−^ in the cytosol, Rubisco-containing carboxysome, and CA. Intracellular pH is buffered via Ca^2+^/H^+^ antiporter, which alone releases OH^−^ in the extracellular environment and increases pH, favoring extracellular CaCO_3_ precipitation. Adapted from [124,138,140,142].

Carbonic anhydrase (CA; EC 4.2.1.1; zinc-containing metalloenzyme) is an enzyme-driven metabolic pathway essential for carbon sequestration [34,93]. The enzyme catalyzes the reversible hydration of CO_2_ (Equation (2)) and plays a role in pH regulation [120]. The enzyme is very complex, containing five distinct classes (α, β, γ, δ, and ε; evolutionarily independent) [131], and α-CA contains 15 isozymes [132]. The enzyme can be intracellular, intra-organellar, periplasmic, or extracellular [120]. Intracellular CA can possess a carbon-concentrating mechanism (CCM) to capture and sequester CO_2_ [131,133,134]. Inorganic-carbon-concentrating mechanisms (Figure 5) can occur from (i) diffusion or active transport of carbon (CO_2_ or HCO_3_^−^) across the cytoplasmic membrane via energy-dependent transporters; (ii) CA conversion to HCO_3_^−^, which accumulates in the carboxysome; (iii) conversion of HCO_3_^−^ back to CO_2_ by CA, which concentrates and fixes elevated concentrations of CO_2_ in the Rubisco [125,133]. The CCM is thought to have evolved from the decline of atmospheric CO_2_ and increase in O_2_ in the Phanerozoic era, triggering oxygenic photosynthesis in cyanobacteria [135]. The CCM genes (ccmK, ccmL, ccmM, ccmN, and ccmO) enable growth at low pCO_2_ for assembly in the carboxysome [125]. The carboxysome (sub-cellular compartment encapsulating Rubisco and CA) is a primary component of CCM [136]. There exists a differentiation between α-cyanobacteria and β-cyanobacteria based on the type of carboxysome and Rubisco [135]. The carboxysome is separated into α-carboxysome and β-carboxysome, both of which limit CO_2_ leaching; reduce the risk of photorespiration; and enhance carboxylase, the activity of Rubisco [136]. The CA enzyme is specific to the type of carboxysome: α-carboxysome requires β-CA (CsoSCA), and β-carboxysome requires β-CA (CcaA) and γ-CA (CcmM) [136,137], but β-CA has a direct involvement in CCM of β-cyanobacteria [135]. β-cyanobacteria also contain non-carboxysomal CA localized in the cell membrane or periplasmic space: α-CA (EcaA) and β-CA (EcaB) [136]. Cyanobacteria have been identified in hot/cold, alkaline/acidic, marine, freshwater, saline, terrestrial, and symbiotic environments [138]. The CCM of α-cyanobacteria and β-cyanobacteria is dependent on their environmental conditions (i.e., pH, carbon content, salinity, temperatures, oxygen content, light, wet/dry conditions) [138]. However, pH is most prominent since it is linked to carbon speciation (i.e., H_2_CO_3_, CO_2_, HCO_3_^−^, CO_3_^2−^) [136].

However, α-CA are localized in the periplasmic or extracellular space, and it is hypothesized that they are able to convert diffused CO_2_ into HCO_3_^−^ for bacterial metabolism [139]. Extracellular α-CA has been identified from prokaryote *Pseudomonas fragi* [140], *Bacillus* sp. [131], cyanobacteria *Microcoleus chlthonoplastes* [141], *Bacillus mucilaginosus* [142]. Both intracellular and extracellular CA could be detected in soil bacteria, whereby some CA was absorbed by soil [143]. The extracellular CA likely stabilizes the pericellular pH and induces carbonate precipitation [141] at or near the bacterial cell wall.

**Figure 6:**
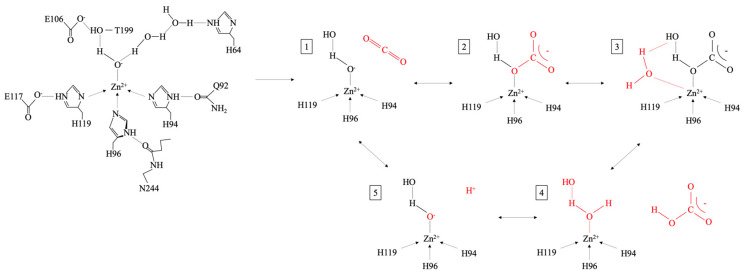
Simplified schematic of the structure-based reaction mechanism of CA. CA II active site structure (**left**) and reaction (**right**). The Zn-bound OH^−^ attacks the carboxyl carbon of CO_2_ (1), creating Zn-bound HCO_3_^−^ (2). A water molecule (3) replaces the HCO_3_^−^ bound to Zn (4), and H^+^ is transferred to solution (5). Adapted from [146,150,151].

While nickel is at the core of urease, CA contains a zinc core [144]. The zinc plays a vital role in the CA activity [145]. There are several genetically distinct forms of CA, each containing a catalytically obligatory zinc ion (Zn^2+^) [146]. The hydrogen bonding network stabilizes the electrostatic environment of zinc, impacting catalytic efficacy [147]. The structure of CA (Figure 6) and orientation of CO_2_ enhance the likelihood of nucleophilic arrack of the Zn^2+^-bound water molecule. This leads to HCO_3_^−^ formation, which is subsequently replaced by a water molecule, releasing HCO_3_^−^ from the active site [148]. The addition of zinc as zinc sulfate (ZnSO_4_) can have a positive effect on CA activity [144]. However, studies show that the zinc ion in the enzyme is firmly bound to the protein, forming a stable metal–protein complex, and therefore, ion exchange with Zn^2+^ in solution is unlikely [149]. CA inhibition can occur via bonding to the zinc-coordinated water molecule/hydroxide ion [134]. This can occur via metal complexing anions and substitution of the non-protein zinc ligand [148].

The enzyme-driven pathways (urease and CA) can be independent processes, but they can also work synergistically to facilitate carbon sequestration [54,93,152]. CO_2_ dissolution can decrease pH due to proton enrichment [21,153]. However, there will also be a pH increase from NH_4_^+^ from urea hydrolysis [154], which maintains the alkaline state required for precipitation [93]. Further, the urease enzyme has nickel incorporated in the active center, and an increase in CO_2_ (regulated by CA) is shown to generate ligands for nickel binding, which is essential to urease activity [26,155]. The nickel core is dependent on CO_2_/HCO_3_^−^ metabolism [93]. However, it should be noted that carbon sequestration can occur independently as a purely chemical reaction [33], whereby CO_2_ is dissolved in water, converted to CO_3_^2−^, and then precipitated as CaCO_3_ without biological interference.

## 3. Carbon Sequestration

**Figure 7:**
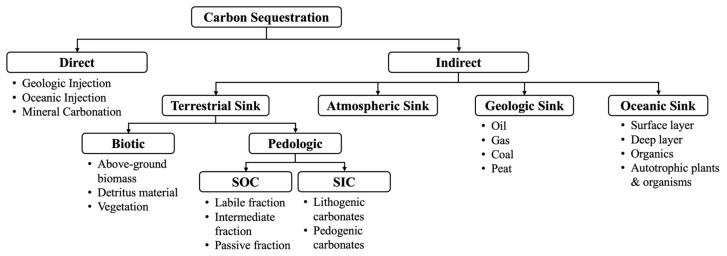
Direct and indirect carbon sequestration methods. Adapted from [40,41,156].

Carbon sequestration methods are characterized as direct (i.e., involved in reduction in CO_2_ emissions by sequestering inorganic carbon prior to atmospheric release) or indirect (i.e., reliance on natural carbon sinks) [41]. The direct methods require CCS techniques for CO_2_ removal, which includes absorption (chemical, physical), adsorption (adsorber beds, regeneration methods), cryogenics, membranes (gas separation, gas absorption, ceramic-based systems), and microbial/algal systems [41]. Mechanisms for carbon sequestration are outlined in Figure 7.

As shown in Figure 7, there are 4 major carbon pools responsible for indirect carbon sequestration. A carbon pool refers to a system that can accumulate or release carbon [157], and these include oceanic, geologic (fossil fuels i.e., coal, oil, gas, peat), terrestrial (pedologic (i.e., SOC, soil inorganic carbon (SIC)) and biotic (i.e., vegetation), and atmospheric pools [40]. A major factor in the global warming crisis is the depletion of fossil fuel carbon pools and the anthropogenic release of GHGs back into the atmosphere. The majority of GHGs are released by the combustion of fossil fuels for energy and transportation. Current methods of carbon sequestration are typically defined as abiotic (oceanic injection, geologic injection, mineral carbonation) or biotic (oceanic sequestration, terrestrial sequestration, mineral carbonation) processes [40].

Oceanic carbon sequestration can be both an abiotic and biotic process. These techniques rely on the solubility pump and autotropic mechanisms [41]. Methods can stimulate growth of autotrophic organisms (phytoplankton, microalgae, macroalgae, and cyanobacteria) on the ocean surface to enhance photosynthesis to remove atmospheric CO_2_ [41]. The methods can utilize DOC for: (i) photosynthesis; (ii) remineralization; (iii) assimilation by microorganisms [158]. The trapped carbon via biological carbon pumps (i.e., gravitational settling, ocean mixing and animal migrations) to mobilize the organic matter downward for burial at the ocean bottom [159,160]. Alternatively, CO_2_ can be injected in liquified phase directly into the deep ocean (> 1 km) to form CO_2_ hydrate for permanent storage [161].

Deep geologic injection, as named, involves deep geologic injection of supercritical CO_2_ into porous aquifers (i.e., coal seams, oil beds, deep saline aquifers) capped by low permeability rock [162]. The CO_2_ can be trapped by: (i) mineral trapping through precipitation of carbonate minerals; (ii) geologic trapping by physical containment in geologic features; (iii) solubility trapping via dissolution in liquid; (iv) hydrodynamic trapping from CO_2_ and liquid viscosity differences; (v) capillary trapping due to capillary forces; (vi) sorption of CO_2_ on the materials surface [21]; (vii) formation trapping by reduced geologic permeability to reduce CO_2_ leakage [53]. In saline aquifers, the CO_2_ injected in supercritical state can be sequestered hydrodynamically by reacting with dissolved salts forming carbonate minerals, by an additive of lower density. The lower viscosity solution displaces brine, which creates a multiphase (gas-like and aqueous) environment [40]. Sedimentary basins are well suited for CCS via deep geologic injection due to the high pore volume and connectivity [163].

### 3.1. Mineral Carbonation and Carbon Sequestration

Mineral carbonation is significant to both abiotic and biotic carbon sequestration methods. The method can be naturally occurring mimicking the natural weathering process of alkaline silicates (Equations (19)–(21)) [164]. The process dissolves atmospheric CO_2_ in rainwater to process weak carbonic acid (H_2_CO_3_), which is slightly acidic causing metal ions to leach from natural alkaline silicates neutralizing their mineral alkalinity and precipitating of carbonates [164]. The process precipitates geologically, geochemically and thermodynamically stable carbonate precipitates [40,164,165]. These precipitates are low solubility [166], and would require acidic conditions or high temperatures (~900 °C) to release CO_2_ from the mineralized precipitate [167]. Mineral carbonation is therefore considered permanent solution for carbon sequestration of atmospheric CO_2_ [168]. Carbonation is impacted by: solid to liquid ratio, particle size, temperature, ion transport mechanisms [166], pH, crystal ageing, agitation, and impurities [169]. It should be noted that environments with high pH leachate from weathering with high CaCO_3_ precipitation can: (i) smother benthic ecosystems; (ii) damage littoral aquatic habitats; (iii) reduce light penetration to benthic primary producers; and (iv) harm fish populations [170].

(19) $$CaSiO_{3(s)}+2{CO}_{2(aq)}+H_{2}O_{\left(l\right)}\to {{Ca}^{2+}}_{\left(aq\right)}+2{{HCO}_{3}^{-}}_{\left(aq\right)}+SiO_{2(s)}$$

(20) $$MgSiO_{4(s)}+4{CO}_{2(aq)}+2H_{2}O_{\left(l\right)}\to 2{{Mg}^{2+}}_{\left(aq\right)}+4{{HCO}_{3}^{-}}_{\left(aq\right)}+SiO_{2(s)}$$

(21) $$({{Mg}^{2+},{Ca}^{2+})}_{\left(aq\right)}+{{CO}_{3}^{2-}}_{\left(aq\right)}\to (Mg,Ca){{CO}_{3}}_{\left(aq\right)}\to (Mg,Ca){{CO}_{3}}_{\left(s\right)}$$

Accelerated carbonation is a process used to replicate the natural weathering process. It speeds up the process by utilizing high-purity CO_2_, which reacts with alkaline materials in the presence of moisture to precipitate carbonates within minutes or hours [171]. There are two main categorizes of processes: the direct method (single reaction step) and the indirect methods (alkaline metal ions are extracted prior to carbonate precipitation in a multi-step process) [164,165,172]. A primary advantage of indirect processes is the production of pure CaCO_3_ (or MgCO_3_) without impurities (i.e., silica) [173]. The process can further be classified as in-situ or ex-situ approaches [174,175]. The former injects CO_2_ directly into the porous material to react with the host rock, whereas the latter uses industrial chemical processes to carbonate natural minerals and industrial waste in treatment plants [174]. Interestingly, the use of alkaline waste originates from industrial and mining activities as host rock for carbon sequestration [165,166,172,176], whose operations and feedstock are often located near point-source GHG emission sources [175]. The operations are promising for storage of atmospheric CO_2_ as carbonate precipitates and offsetting CO_2_ emissions from high-GHG producers. Natural CO_2_ sequestration has been demonstrated in chrysotile mine tailings in Clinton Creek, YT and Cassiar, BC, whereby the process is accelerated by the increased surface area from milling [177]. Researchers have also incorporated CA into accelerated mineral carbonation of alkaline brucite (Mg(OH)_2_) to overcome the carbonation rate-limiting supply of CO_2_, demonstrating acceleration of 240% over controls [178].

Direct mineral carbonation methods (Table 3) can be gas-solid carbonation or aqueous carbonation (gas-liquid or gas-liquid-solid) [164]. The operation is simple relying on an input of CO_2_ to facilitate precipitation of carbonate minerals. Direct precipitation can occur under dry or moist conditions [164]. However optimal CO_2_ sequestration often requires a degree of moisture [179,180,181,182]. The particle size also plays an important role, whereby smaller particle sizes are preferable [180,183]. Mechanical pretreatment (crushing and grinding) can be used to reduce particle size <300 μm, destroying the mineral lattice and increasing surface area for the reaction [175]. Researchers are also studying thermal pre-treatment, NETL derived processes, brine-based processes, and organic acid direct processes [175]. Although these processes can be considered indirect stepwise gas-solid methods [184]. Fagerlund et al. [185], for example, are studying stepwise carbonation of serpentinite (Equations (22) and (23)) at Åbo Akademi University, whereby magnesium ions are released from Mg_3_Si_2_O_5_(OH)_4_ through heat (400–500 °C) and ammonium sulphate ((NH_4_)_2_SO_4_) induce precipitation of magnesium sulfate (MgSO_4_), which afterwards is used to precipitate magnesium hydroxide (Mg(OH)_2_) via an aqueous ammonium hydroxide solution (NH_4_OH). The Mg(OH)_2_ precipitate is then used for carbonation with CO_2_ injection to precipitate MgCO_3_.

(22) $${Mg}_{3}{Si}_{2}O_{5}{\left(OH\right)}_{4}+3{{(NH}_{4})}_{2}{SO}_{4}\to 3Mg{SO}_{4}+2{SiO}_{2}+5{H_{2}O}_{\left(g\right)}+6{NH}_{3(g)}$$

(23) $$Mg{SO}_{4}+2{{NH}_{4}OH}_{\left(aq\right)}\to {\left({NH}_{4}\right)}_{2}{SO}_{4(aq)}+Mg{\left(OH\right)}_{2}$$

Alternatively, the direct aqueous method relies on the reaction of CO_2_ and water (Equation (2)) to form HCO_3_^−^ and a proton (H^+^), which releases the divalent cation from the mineral (Equation (24)) to precipitate carbonate (Equation (25)) [199]. The reactions can be accelerated more via additives (i.e., bicarbonate/salts, acids, or chemical activators) or pretreatment methods (comminution, magnetic separation, heat treatment), which alter reaction conditions and modify solution chemistry to increase reaction rates to increase carbonate precipitation [172].

(24) $$MgSiO_{4}+4H^{+}\to 2{Mg}^{2+}+SiO_{2}+2H_{2}O$$

(25) $${Mg}^{2+}+2{HCO}_{3}^{-}\to MgCO_{3}+H^{+}$$

Indirect mineral carbonation typically utilizes stepwise gas-solid, pH swing, or chemically enhanced mechanisms [184]. In the former, acid addition is utilized to enforce metal separation and a base additive induces aqueous carbonation [164,184]. The latter, chemically enhanced mechanisms, can include: HCl extraction, the molten salt process, other acid extractions (acetic acid (CH_3_COOH), sulfuric acid (H_2_SO_4_), nitric acid (HNO_3_), and formic acid (HCOOH)), bioleaching, ammonia extraction, and caustic extraction [172]. Acid extraction merely extracts the desirable metals from mineral prior to aqueous carbonation [164], causing potentially unfavorable conditions for precipitation (i.e., acidic conditions cause low CO_2_ dissolution and the low pH inhibits precipitation) [172]. HCl extraction, for example, showed no precipitated CaCO_3_ due to the rapid pH decline causing acidic conditions inhibition precipitation [200]. The pH swing process was developed by Park and Fan [201] to overcome these limitations. Wang and Maroto-Valer [202] have developed an amended pH swing method for serpentine incorporating the following steps: (i) CO_2_ is captured and reacts with NH_3_ to form NH_4_HCO_3_ intermediary; (ii) mineral dissolution via NH_4_HSO_4_ additive producing MgSO_4_; (iii) NH_4_OH is added to neutralize pH and remove impurities; (iv) MgSO_4_ reacts with NH_4_HCO_3_ at mild temperature to form Mg(HCO_3_)_2_ converting in the presence of water to MgCO_3_; (v) recovery of (NH_4_)_2_CO_3_ from carbonation via evaporation and heating to produce NH_4_HSO_4_ and NH_3_ for reuse.

Bioleaching utilizes byproducts (i.e., production of organic acids, chelating and complexing compounds) excreted by microorganisms to extract metals from minerals [203]. The humic and organic acids, inorganic acids, and chelating agents can free nutrients enhancing physical and chemical weathering [204]. These steps can be categorized by direct (Equation (26)) or indirect (Equation (27)) bacterial leaching, whereby both equations show metal sulfide (MS) oxidation into metal sulfate (MSO_4_) [203]. In Equation (26), bacterial enzyme activity catalyzes the mineral sulfate oxidation through direct physical contact between the bacterial cell and the mineral sulfide surface [203]. Metal sulfides include: covellite (CuS), chalcocite (Cu_2_S), sphalerite (ZnS), galena (PbS), molybdenite (MoS_2_), stibnite (Sb_2_S_3_), cobaltite (CoS), millerite (NiS), and pyrite (FeS_2_) [203]. The indirect oxidation in Equation (27), is generated by a catalytic function of a lixiviant which chemically oxidizes the sulfides [203]. In both scenarios, potentially acid generating substances (i.e., comprising sulfides or elemental sulfur) provide food for bacteria, which generated sulfuric acid as a by-product of metabolism, which leaches metals from minerals [172]. Furthermore, autotrophic bacteria (i.e., chemolithoautotrophic) can fix carbon biologically through the process by utilizing inorganic, atmospheric CO_2_ instead of organic carbon for new cell synthesis [172,203]. Factors influencing bioleaching include nutrients, O_2_ and CO_2_ content, pH (acidic conditions), temperature, mineral substrate (dependent on mineralogical composition and particle size), heavy metals, surfactants and organic extractants (decrease surface tension and mass transfer or oxygen) [203]. Chiang et al. [205] attributed bioleaching to organic acid production, specifically gluconic acid (C_6_H_12_O_7_), and microbial exopolysaccharides (EPSs). Similar to other mineral carbonation techniques, the released metal ions are available for carbonate precipitation. Bioleaching of ultramafic mine tailings can be used at tailings storage facilities [206]. Chrysotile tailing indicate acid mine drainage environments with microbial catalyst from *Acidithiobacillus* sp. is promising for MgCO_3_ precipitation with atmospheric CO_2_ resulting in 316 kt Mg leached/10 Mt tailings (458 kt CO_2_ sequestered/year) [207]. Argon Oxygen Decarburization slag showed a decline in primary phase (dicalcium-silicate, bredigite, and periclase) and an increase in secondary phases (merwinite and calcite), specifically a 3.1 wt % increase in CaCO_3_ with *B. mucilaginous* bacterial species [205].

(26) $$MS+2O_{2}\overset{Bacteria}{\to }M{SO}_{4}$$

(27) $$MS+{Fe}_{2}{\left({SO}_{2}\right)}_{3}\to M{SO}_{4}+2Fe{SO}_{2}+S^{0}$$

### 3.2. Advancements to Mineral Carbonation for Carbon Sequestration

Microbial carbon mineralization has a unique opportunity to utilize novel biochemical mechanisms for carbon sequestration. The process can sequester inorganic carbon as a CCS technique via carbonate precipitation, but also mitigate CO_2_ released to the atmosphere therefore reducing atmospheric CO_2_ levels to aid the impacts of climate change [208]. The MICP enzyme-driven reactions speed up chemical reactions to enhance the rate of reaction and therefore conversion to CaCO_3_, optimizing storage of inorganic CO_2_. The synergistic role of CA and urease enzymes work to hydrate atmospheric CO_2_ while inducing an alkaline state for biocalcification [93]. Again, alkaline material is a preferential substrate for carbon sequestration [165,166,172,176], therefore halotolerant and alkalophilic bacteria are required for biocalcification. Ureolytic bacteria offer a preferable metabolic pathway for MICP, since microorganisms have shown capable of withstanding unfavorable conditions for bioremediation [154,209,210], and operate under high pH values and high concentrations of inorganic salts (i.e., CaCl_2_) [211]. Montmorillonite-coupled MICP in cyanide tailings showed up to a 1.33 increase in precipitation and up to 34.55% CO_2_ capture [212]. Other natural environments that supply cations for carbonation include: evaporate deposits, saline aquifers, waste brines, wastes from oil extraction, seawater [213]. MICP has shown success in precipitating carbonate in mine waste [73,154,214,215,216], concrete and building materials [217,218,219,220], coastal and marine environments [38], and agriculture and soil [47,221,222,223], all of which indirectly demonstrate the promising potential for carbon sequestration utilizing various substrates. Table 4 summarizes research investigating MICP as a method for carbon sequestration.

In addition to trapping CO_2_ as mineral precipitates, there exists addition mechanisms in which CO_2_ can be trapped and therefore stored. MCP and MICP have been utilized to aid CCS methods. During deep geologic injection the CO_2_ remains in supercritical state resulting in a distinct phases separate from formation water or brine, which is less dense and viscous permitting CO_2_ leakage [162]. Ureolysis and biofilm formation have been used to enhance mineral trapping, solubility trapping, and formation trapping of supercritical CO_2_ for geologic carbon sequestration [46,53,153,162,233]. Mineral trapping by precipitation of stable carbonates in deep geologic structures can store carbon but also reduce structural permeability to mitigate CO_2_ leakage [234]. Transition-state calcite and siderite have formed in deep saline aquifers by indigenous microorganisms [153]. The formation of biofilms in high-pressure pore spaces can decrease permeability by >95% trapping gaseous CO_2_ [46]. Furthermore, a pH increase generated by urea hydrolysis can increase DIC thereby lowering CO_2_ gas in the headspace for solubility trapping [233].

**Figure 8:**
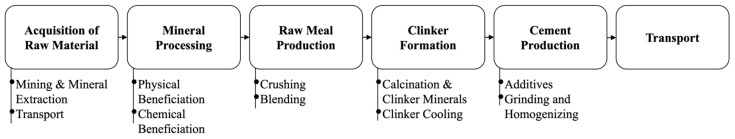
Cement production and the GHG emissions associated with specific processes. Adapted from [236,237].

While carbonate precipitation can directly trap and store atmospheric CO_2_, it can also reduce GHG emissions released to the atmosphere. The cement industry is notorious for its significant contribution to the release of GHG emissions, accounting for 7% of the global GHG emissions and 1.5% (11.2 Mt in 2019) of Canadian emissions [235]. The production of cement (Figure 8) includes mineral extraction of raw materials, mineral processing, raw meal production, clinker formation, cement production and transport [236]. The process produces significant GHG emissions by: (i) calcination reactions (i.e., clinker process; Equation (28)); (ii) combustion of carbon heavy materials (i.e., coal, fuel, natural gas, petroleum coke, etc.); (iii) high energy requirement (2% global energy consumption); (iv) scale of production; (v) material treatment (grinding, mixing, additives); (vi) transport [237]. Self-healing bioconcrete, for example, uses autogenic (chemical reaction precipitating CaCO_3_ from cement hydration) and autonomic processes (application of encapsulation or a continuous vascular system to distribute a healing agent to precipitate CaCO_3_; can include MICP) as advancements to concrete repair [219,220]. MCP and MICP can be used as an alternative method for soil and concrete strengthening and restoration of calcareous stones and construction materials [45], reducing the usage of heavy GHG-producing cements. By precipitating CaCO_3_ or other MCO_3_ compounds in the pore space, cracks or fissures, MCP and MICP creates a clogging effect which strengthens the material and reduces compressibility [223]. This is in conjunction with the formation of a biocement matrix, whereby “preferential” and “uniform” distribution of precipitates at particle-particle contacts and precipitation around solid particles respectively, which improves engineering properties [223]. Application of MICP in cementitious materials for the construction industry showed a decrease in CO_2_ (3800 ppm to 820 ppm) with precipitated calcite and vaterite crystals through recombinantly produced CA MICP [225]. This shows MICP ability for carbon sequestration and carbon negative cementitious materials [225].

(28) $${CaCO}_{3}\to CaO+{CO}_{2}$$

Finally, MCP and MICP can treat environmental disasters caused by climate change. Increased drought and more severe storms are widely accepted effects of climate change. Earthquake-induced liquefaction causes soil to behave fluidlike because of increases in pore water pressure and decreases in effective stress [238]. MICP has shown promising for treatment of liquefiable saturated soils in-situ [239]. MICP can also be used to treat landslide disasters by strengthening sliding surfaces [240]. Furthermore, both deep flooding and heat stress in soil reduced the relative abundance of genes encoding lignin-degrading catalase in *Actinobacteria*, which resulted in increased organic carbon sequestration [241]. Conversely, drought caused by severe water loss via evaporation can be mitigated with MICP through the formation of a surface crust, remediation of desiccation cracks, smaller pore size, and residual solutes, which decrease the rate of evaporation and water loss by reducing water flow through the material [242].

## 4. Future Research

While the topic of bacterial carbonatation is not novel, limited research exists on the use of MICP for carbon sequestration. The following is required to better understand microbial carbon sequestration and its long-term feasibility as a CCS technique for the changing environment:

- Comparisons of MICP utilizing alternative bacteria species to induce different metabolic pathways for the assessment of optimal carbon sequestration.
- Suitability of specific bacterial species for use with different material types to establish conducive environmental conditions for their metabolic pathways and activity. To date, most MICP research evaluates its usage with soil. However, additional research is required regarding alternative materials that are less hospitable environments for microorganisms to determine the practicality of biochemical carbon sequestration near GHG point-source emissions.
- Analysis of biochemical alterations for enhancement and optimal use of enzyme-driven metabolic pathways. In addition to optimal growth conditions for bacteria, which is regularly incorporated into biochemical analyses, an evaluation of chemical additives and their impact on the efficacy of metalloenzymes (i.e., Ni and Zn) with the objective of carbon sequestration.
- Evaluation of MICP and CO_2_ injection to better understand preferable CO_2_ phases (liquid, gas, supercritical state) for biocalcification and pressures microorganisms can withstand to maximize the rate-limiting CO_2_ supply for carbonate precipitation, while minimizing damage to bacterial cells, biomass concentration, and the organic matrix.
- Comprehensive assessment of bacterial carbonation and its impact on precipitate composition, morphology, and stability for long-term storage of inorganic carbon. Impacts at the micro-particle scale and the large-scale feasibility of carbonate precipitation, considering MICP application and its impact on carbonate stability.
- Life-cycle assessments of the MICP process comparing different MICP application methods (i.e., in situ biostimulation, ex situ biostimulation, bioaugmentation, amended bioaugmentation) with traditional carbon sequestration techniques to determine quantitively the carbon emissions vs. carbon sequestered from “cradle” to “grave”.
- Evaluation of the long-term feasibility of MICP with the changing environment due to climate change. The geophysical and biochemical environmental changes (temperature, groundwater conditions, etc.) attributed to climate change and their impacts on specific bacterial species and community diversity, their metabolic activity, and their ability to precipitate carbonates.

MCP and MICP are promising biochemical advancements to the field of carbon sequestration. Research objectives specific to carbon sequestration and CCS are required to further advance the biocalcification methods. Laboratory-scale experimentation and modeling techniques can provide essential information for the development of optimal conditions to maximize carbonate precipitation and the feasibility of combatting climate change.

## 5. Conclusions

Mineral carbonation is an effective method to store atmospheric CO_2_ as mineral carbonates via mineral trapping. The mechanism is facilitated by numerous biogenic and abiogenic precipitation techniques with varying degrees of microbial control. Enzyme-driven MICP utilizing CA and urease synergistically is promising for catalysis of CO_2_ hydration and increase in pH and DIC, leading to increased CaCO_3_ precipitation. Since increases in CaCO_3_ precipitation are directly linked to increases in carbon sequestration, optimization of microbial metabolic activity can favorably impact CaCO_3_ and, therefore, carbon sequestration. Furthermore, solubility trapping and formation trapping via MICP during deep geologic injection of CO_2_ can mitigate CO_2_ leakage. Biological carbonate precipitation is promising for atmospheric carbon sequestration with the changing climate. Addition experimental research is required to evaluate the reliability of the method.

## Figures and Tables

**Table 3:** Direct methods for mineral carbonation and carbon sequestration.

Method	Material	CO_2_ Application	CO_2_ Input ^1^	Results ^1^	Findings	Reference
Direct Gas–Solid Carbonation	Municipal Solid Waste Incinerator	CO_2_ flow	100% CO_2_, 3 bars, 2.5 h	3.19% CaCO_3_ gain bottom ash 7.31% CaCO_3_ gain fly ash	More suitable to small particle size.	[183]
			100% CO_2_, 3 bars, 3 h	11% CaCO_3_ gain fly ash	Optimal CO_2_ capture at water/solid ratio 0.3.	[179]
			17 bars, 3 h	3% CaCO_3_ gain bottom ash	Optimal CO_2_ capture 20% *w*/*w* moisture and 4 mm sieving.	[180]
			1 bar, 1 h	60 g CO_2_/kg fly ash	Temperature (600 °C) and H_2_O_(g)_ (20%) are more significant than CO_2_ content.	[181]
	Waste Concrete & Anorthosite Tailings		18.2 vol% CO_2_, 4 & 5 bar, 30 min	66% CO_2_ removal waste concrete 34% CO_2_ removal anorthosite	Aqueous phase carbonation resulted in 34.6% removal in 15 min.	[186]
	Pre-treated EAF steel-making bag house dust		3 bar inlet, 1 bar (outlet), 12 L/min	0.657 kg CO_2_/kg dust	Carbonation was based on the total calcium content.	[187]
	Air Pollution Control Residues from a Medical Solid Waste Incinerator		100% CO_2_, 6 h	0.12 kg CO_2_/kg dry solid waste	Maximum carbonation at 400 °C.	[188]
	Serpentinite Mining Residue	CO_2_ concentration	18 vol% CO_2_	0.07 g CO_2_/g residue	Water vapor (10 vol%) required for carbonation.	[182]
Direct Aqueous Carbonation	Concrete Fines	CO_2_ flow	14% CO_2_, 90 min	0.19 g CO_2_/g concrete fines	Almost all absorbed CO_2_ was converted to CaCO_3_, and increased CO_2_ concentration requires higher solid–liquid ratio.	[189]
	Olivine with NaHCO_3_ & NaOH Buffers		pCO_2_ 6.5 MPa, 6 h	<80% carbonation	Agitation is necessary to prevent solids settlement. Low pCO_2_ requires high NaHCO_3_ concentration.	[190]
	Flue Gas Desulfurization Gypsum		1 L/min, 15 min	90% CaCO_3_ efficiency	CaCO_3_ precipitation increased linearly with ammonia content.	[191]
	Steel Slag		19 bar CO_2_, 30 min	0.25 kg CO_2_/kg steel slag	Primary factors: particle size <2mm to <38 μm and temperature 25–225 °C.	[192]
	Red-Mud		3.5 bar, 3.5 h	5.3 g CO_2_/100 g red mud	At liquid–solid ratio of 0.35.	[193]
	Oil Shale Ash		Continuous flow (0.7 m/10 L), 15% CO_2_	17–20% bound CO_2_	Size and structure of CaCO_3_ depended on end-point pH.	[194]
	Coal Fly Ash		10 bars, 18 h	26 kg CO_2_/ton fly-ash	Pressure was independent of carbonation efficiency and not affected by temperature of fly ash weight.	[195]
	Industrial/Mining Wastes	CO_2_ concentration	15% CO_2_	544.6 g CO_2_/kg carbide slag	Ca content in material produces increased carbonation. Max carbon sequestration occurred at < 75 μm particle size, 60 °C, 100 g/L liquid–solid ratio.	[196]
	Aggregate Recycling Concrete Fines		5% CO_2_	0.13 g CO_2_/g concrete fines	0.10 CO_2_/g concrete fines captured as CaCO_3_, and 0.02 CO_2_/g concrete fines dissolved in aqueous.	[197]
	Low-Calcium Fly Ash		30% CO_2_	0.016 g CO_2_/g fly ash	Good carbonation potential despite low energy input and low calcium content.	[198]

^1^ As reported in the literature.

**Table 4:** Carbon sequestration using MICP.

Metabolic Pathway	Microbial Strain	Material	Findings	Reference
CA	*Citrobacter freundii*	Wastewater	CaCO_3_ precipitated with CO_2_ catalyzed by CA. Can sequester CO_2_ at high concentrations, but HCO_3_^−^ inhibits CA enzyme activity due to pH decrease.	[224]
	*Bacillus subtilis*	Agar & Liquid Medium	CA converted CO_2_ to CaCO_3_ minerals.	[225]
	*Bacillus cereus*	Karst Soil	CA enzyme activity influenced CaCO_3_ crystal morphology.	[226]
	*Bacillus megaterium*	Mortar Specimens	CO_2_ influx precipitated comparable CaCO_3_ to ureolysis-precipitated CaCO_3_	[114]
	*Bacillus pumilus*, *Bacillus marisflavi*	Seawater	$\mathrm{CA}\;\mathrm{was}\;\mathrm{observed},\;\mathrm{and}\;\mathrm{precipitates}\;\mathrm{included}\;{\mathrm{CaCO}}_{3}\cdot$H_2_O and CaCO_3_, showing the potential for carbon sequestration.	[227]
	*Bacillus altitudinis*	Mangrove Soil	Impact of CO_2_ sequestration with bacteria showed 75% removal and 97% removal with bacteria and CA.	[144]
	*Bacillus mucilaginosus*	Liquid Medium	Optimal CA at 30 °C and alkaline environment to enhance CO_2_ hydration.	[228]
	*Bacillus mucilaginosus*	Liquid Medium	CO_2_ is more easily captured by CA, which alters the size and morphology of CaCO_3_ crystals.	[229]
	*Psychrobacter* sp., *Vibrio alginolyticus*	Marine Sediments	Strong potential for carbonate precipitation with high CA, meaning capture of CO_2_.	[230]
EPS & CA	*Bacillus cereus*	Liquid Medium	Calcite induced by bacteria can fix CO_2_ from air since CO_2_ released from organic matter is less than in air.	[231]
	*Curvibacter lanceolatus*	Liquid Medium	CA precipitated only calcite, whereas CA and EPA precipitated calcite and aragonite to enhance CO_2_ fixation.	[232]
Phototrophic	*Phragmoplastophyta*	Diamond Mine	Secondary carbonate precipitation capable of offsetting CO_2e_ by 20%.	[129]
	*Oscillatoria* sp., *Porphyrobacter* sp., *Blastomas* sp., *Rhodobacter* sp.	Diamond Mine	Kimberlite weathering and secondary carbonate precipitation can sequester carbon through photosynthetic bacteria acting as a catalyst to convert CO_2_ to CaCO_3_/MgCO_3_.	[130]
Ureolysis	*Sporosarcina*, *Sphingobacterium*, *Stenotrophomonas*, *Acinetobacter*, *Elizabethkingia*	Cave & Tavern Water	CO_2_ sequestration depended on pH and the consortia of bacteria.	[113]
	*Sporosarcina*, *Brevudimonas*, *Sphingobacterium*, *Stenotrophomonas*, *Acinetobacter*	Cave & Tavern Water	Abiotic CO_2_ sequestration depended on pH and medium, whereas biotic CO_2_ sequestration depended on the bacterial species or strains.	[33]
	*Sporosarcina pasteurii*	Tailings	MICP increased CO_2_ capture from tailings by 27.15–34.55%	[212]

## Data Availability

Data are contained within the article.
